# Magnesium Phosphate Cement as Mineral Bone Adhesive

**DOI:** 10.3390/ma12233819

**Published:** 2019-11-21

**Authors:** Theresa Brückner, Markus Meininger, Jürgen Groll, Alexander C. Kübler, Uwe Gbureck

**Affiliations:** 1 Department for Functional Materials in Medicine and Dentistry, University Hospital Würzburg, Pleicherwall 2, 97070 Würzburg, Germany; brueckner.theresa@gmail.com (T.B.); markus@4meininger.de (M.M.); juergen.groll@fmz.uni-wuerzburg.de (J.G.); 2 Department of Oral & Maxillofacial Plastic Surgery, University Hospital Würzburg, Pleicherwall 2, 97070 Würzburg, Germany; kuebler_A@ukw.de

**Keywords:** magnesium phosphate cement, phytic acid, bone adhesive

## Abstract

Mineral bone cements were actually not developed for their application as bone-bonding agents, but as bone void fillers. In particular, calcium phosphate cements (CPC) are considered to be unsuitable for that application, particularly under moist conditions. Here, we showed the ex vivo ability of different magnesium phosphate cements (MPC) to adhere on bovine cortical bone substrates. The cements were obtained from a mixture of farringtonite (Mg_3_(PO_4_)_2_) with different amounts of phytic acid (C_6_H_18_O_24_P_6_, inositol hexaphosphate, IP6), whereas cement setting occurred by a chelation reaction between Mg^2+^ ions and IP6. We were able to show that cements with 25% IP6 and a powder-to-liquid ratio (PLR) of 2.0 g/mL resulted in shear strengths of 0.81 ± 0.12 MPa on bone even after 7 d storage in aqueous conditions. The samples showed a mixed adhesive–cohesive failure with cement residues on the bone surface as indicated by scanning electron microscopy and energy-dispersive X-ray analysis. The presented material demonstrated appropriate bonding characteristics, which could enable a broadening of the mineral bone cements’ application field to bone adhesives.

## 1. Introduction

There are two main indications for the application of bone adhesives: one is to joint bone fragments of comminuted fractures as a bone-to-bone interface. This allows the partial substitution of fracture fixation by metal components such as wires, screws, and plates especially for small fragments with a size of < 1 cm [1]. Furthermore, bone adhesives can serve as an additional bone-to-metal interface for metallic alloys [2]. An application for such indications would require appropriate adhesion to both bone as well as metal compartments. In particular, in the first case, the adhesive material must not impair fracture healing by forming a rigid barrier, which is enabled while applying the bone adhesive only selectively or by using biodegradable materials. Recent approaches to create bone adhesives include cyanoacrylates [3,4], methacrylates [5,6,7], fibrin glue [8], mussel proteins and dopamine hydrogels [9,10,11], or polysaccharides [12,13,14]. Most synthetic materials that have been tested as bone-bonding agents, are either mechanically weak with insufficient adhesion to the bone surface or do not fulfill the requirement of biodegradability, as for example poly(methyl methacrylate) (PMMA), cyanoacrylates and its associated formulations [15].

In contrast, mineral bone cement formulations based on calcium and magnesium phosphate chemistry (CPC and MPC) are biodegradable in vivo [16,17], but were actually not developed for the application as bone adhesives. In particular CPC are considered to have relatively low bone adhesiveness [15], why most research focused on the implementation of calcium phosphate fillers into methacrylated polymer-based composite systems [18,19,20]. Compared to CPC, some of the material characteristics of MPC are outstanding, which includes their high initial strength [21], a more reliable degradation potential [16,17] and they are considered to have a higher bone affinity [15,22].

In the present study, we chose a MPC in which farringtonite (Mg_3_(PO_4_)_2_) reacted with phytic acid (C_6_H_18_O_24_P_6_, inositol hexaphosphate, IP6) via a chelation mechanism to form a stable complexed magnesium phosphate cement [23]. During our previous work on this system we observed a high adhesiveness of this cement type to the surfaces of tools such as glass slabs, spatula, or molds. This was further explored in the current study to cortical bone substrates to determine the adhesiveness ex vivo in a shear test regime. We hypothesized that the phytic acid will be the reason for the cement formulation to show a higher bone affinity compared to conventional mineral bone cements, as phytic acid is able to form insoluble complexes with calcium [24,25] and magnesium cations [20], which are contained in the raw powder (Mg) as well as in the used bone slices [26].

## 2. Materials and Methods

### 2.1. Cement Preparation

0.6 mol MgHPO_4_·3H_2_O (newberyite) was mixed with 0.3 mol Mg(OH)_2_ and tempered for 5 h in a sintering oven (Oyten Thermotechnic, Oyten, Germany) at 1100 °C. The sintered cake was crushed, sieved < 355 µm and milled in a planetary ball mill (Retsch, Haan, Germany) for 1 h at 200 rpm. The resulting raw powder Mg_3_(PO_4_)_2_ (farringtonite) was compounded with different amounts of MgO Magnesia 2933 (6.0 wt.%, 6.8 wt.% or 7.5 wt.%; Magnesia, Lüneburg, Germany) and mixed with variably concentrated phytic acid solution (C_6_H_18_O_24_P_6_, 20.0%, 22.5% or 25%) in a powder-to-liquid ratio (PLR) of 2.0 g/mL. Low MgO amounts were combined with low phytic acid concentration and vice versa. Mg_3_(PO_4_)_2_ was blended with 2.0 M phosphoric acid (H_3_PO_4_, Merck, Darmstadt, Germany) with a PLR of 2.0 g/mL for reference measurements. If not else described, all chemicals originate from Sigma Aldrich, Steinheim, Germany.

### 2.2. Compressive Strength Testing

The cement pastes were transferred into silicone rubber molds (6 × 6 × 12 mm^3^), stored for 1 h at 37 °C and > 90% humidity, demolded, covered with phosphate buffered saline (PBS, 8.0 g/L NaCl, 1.1 g/L Na_2_HPO_4_, 0.2 g/L KCl, 1.1 g/L KH_2_PO_4_; potassium salts purchased from Merck, Darmstadt, Germany, and sodium salts purchased from Sigma Aldrich, Steinheim, Germany; pH = 7.4) and stored at 37 °C. The wet compressive strengths of the cuboids (n = 12) was measured 24 h respectively 7 d after fabrication. In the latter case, PBS was changed twice. For initial mechanical testing, the samples were directly analyzed after demolding. A universal testing machine Z010 (Zwick, Ulm, Germany) was used at a crosshead speed of 1 mm/min.

### 2.3. Phase Composition

The cement fragments were ground and further analyzed via X-ray diffractometer (XRD) D5005 (Siemens, Karlsruhe, Germany). Cu-K_α_ radiation, a 40 kV voltage, a 40 mA current, a 2 theta range from 20 to 40°, a step size of 0.02° and a scan rate of 1.5 s/step were used. Joint Committee on Powder Diffraction Standards (JCPDS) references were considered for XRD pattern evaluation.

### 2.4. Adhesion Testing

For measuring the cement adhesion to bone, ~3 mm × 10 mm × 20 mm cortical bone samples were prepared from fresh bovine femurs by sawing and grinding their surface with SiC-paper (grit P80, Schmitz-Metallographie, Herzogenrath, Germany). A disc-shaped silicone rubber mold (2 × 5 mm^2^) was fastened centrically onto a bovine bone substrate and filled with the as-prepared cement paste. After 10 min, the samples were demolded such that the cement disc directly stuck on the bone surface. The shear force as a measure for bone adhesiveness (n = 5) was tested via universal testing machine Z010 (Zwick, Ulm, Germany) with a crosshead speed of 1 mm/min in the dry state after fabrication (initial adhesiveness) and after further immersion for 24 h respectively 7 d in PBS at 37 °C. For the 7 d deposition in PBS, the buffer was changed twice. When the samples dropped off before measurement, the shear strength was set to zero, when the samples dropped off because of the net weight (66.23 g) of the stamp, this weight was taken into account for further shear strength calculations. For all other samples, the net weight of the stamp was also added to the maximum force and consequently included in shear strength calculations.

### 2.5. Scanning Electron Microscopy and Energy-Dispersive X-ray Spectroscopy

After having tested the adhesiveness, cement residues were visualized by scanning electron microscopy (SEM) and energy-dispersive X-ray spectroscopy (EDS). The bone slices were dried by immersion in mixtures of acetone with ultrapure water (30, 50, 70, 90 and 6 × 100%) for 1 h respectively, followed by super-critical drying with the device CPD 030 (Bal-Tec, Balzers, Liechtenstein). First, the acetone was exchanged by liquid CO_2_ at a temperature of 6 to 11 °C (10 exchange cycles), followed by an evaporation of CO_2_ at 42 °C. Afterwards, the bone surfaces were sputtered with a 4 nm platinum layer (Leica EM ACE600, Leica Microsystems, Wetzlar, Germany). The morphology of adhesive residues was determined with a scanning electron microscope Crossbeam 340 (SEM, Zeiss, Oberkochen, Germany). Furthermore, EDS was performed with an INCA Energy 350 AzTec Advanced system using a silicon drift detector (SDD) (Oxford Instruments, Abingdon, UK) for element mapping with a resolution of 512, 10 frames, a process time of 4, a pixel retention time of 10 µs, a 60 µm slit and a high voltage of 20 kV. For semi-quantitative evaluations of corresponding Ca-to-Mg-ratios, the percentaged amount of Ca and Mg was calculated based on the border EDS element maps. For each region (cement, adherend), three ellipsoid sectors were analyzed each.

### 2.6. Statistics

Significant differences (p < 0.01) were calculated using a 1-way ANOVA (inerSTAT-a v1.3, M.H. Vargas, Instituto Nacional de Enfermedad Respiratorias, Mexico).

## 3. Results

**Figure 1:**
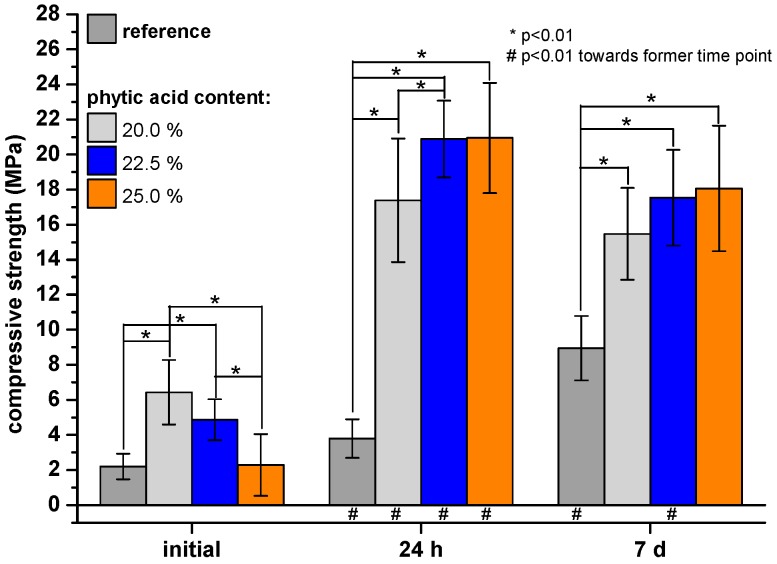
Compressive strength of cement cuboids made from farringtonite and 2.0 M phosphoric acid (reference) respectively 20.0, 22.5 or 25.0% phytic acid initially or after 24 h respectively 7 d hardening in PBS at 37 °C.

We determined the intrinsic material strength via a compressive strength test setup (Figure 1). Obviously, the phytic acid containing samples had a significant higher compressive strength at every measured time point compared to the reference. Only those samples with a 25.0% phytic acid amount had an initial strength after 1 h setting of 2.29 ± 1.76 MPa which was comparable to the reference strength (2.21 ± 1.76 MPa). In the course of 7 d, the reference compressive strength increased by 305% up to 8.95 ± 1.84 MPa, while the phytic acid modified cements had strengths in the range 16–18 MPa.

**Figure 2:**
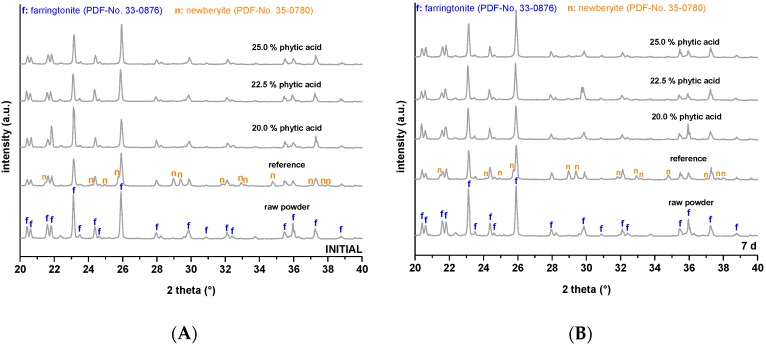
Corresponding XRD patterns initially (**A**) respectively after 7 d (**B**) hardening of the cement prepared at a powder-to-liquid ratio of 2.0 g/mL. Characteristic reflexes are marked with f (farringtonite, PDF-No. 33-0876) respectively n (newberyite, PDF-No. 35-0780) in comparison to JCPDS references.

Corresponding XRD patterns (Figure 2A,B) showed that the reference cement formed newberyite (MgHPO_4_·3H_2_O). Comparing the initial XRD patterns to the patterns after 7 d, quantitative differences could not be seen for phytic acid containing cements and no further crystalline phase has formed.

**Figure 3:**
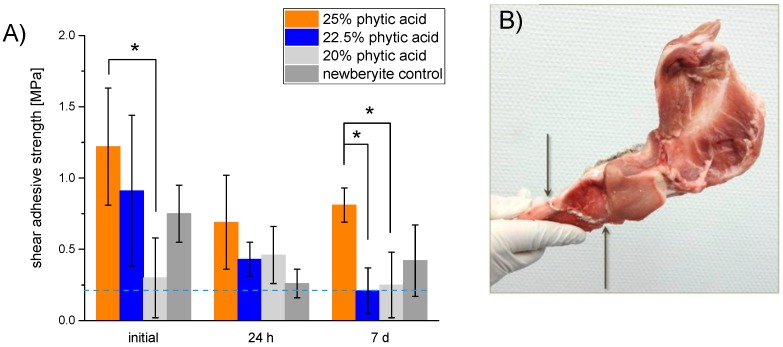
(**A**) Shear strength of cement adhesives made from farringtonite and either 2.0 M phosphoric acid (newberyite reference) respectively 20.0, 22.5 or 25.0% phytic acid initially or after 24 h respectively 7 d hardening in PBS at 37 °C on bovine cortical bone substrates. (**B**) porcine mandible which was manually broken and fixed with 25% phytic acid bone adhesive. The arrows mark the beginning and end of the bonding slit.

Figure 3 reveals the corresponding adhesive strength of various cement formulations on bovine bone. The blue dotted line marks a strength of 0.2 MPa which was determined 1984 by Weber and Chapman [27] to be a threshold value for bone-bonding agents. Adhesives with a lower strength would not be feasible during surgery [27]. Initially, all samples showed an appropriate adhesiveness on bone between 0.75 ± 0.20 (reference) to 1.22 ± 0.41 MPa (25.0%). Samples with the lowest phytic acid content represented an exception, as their adhesiveness laid underneath the as-mentioned threshold value. After ageing, only the composition with a 25.0% phytic acid amount had an appropriate adhesiveness of 0.81 ± 0.12 MPa still after 7 d. With this composition, it was possible to fix a manually broken porcine cadaver mandible such that after 10 min the bonding slit withstood the weight of the heavier part as demonstrated in Figure 3B.

**Figure 4:**
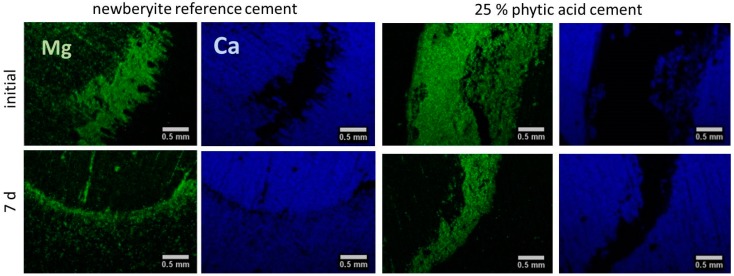
Examples of EDS element maps of cement adhesives made from farringtonite and either 2.0 M phosphoric acid (reference) or 25.0% phytic acid initially or after 7 d hardening in PBS at 37 °C on ground bovine bone samples. All cements were prepared in a powder-to-liquid ratio of 2.0 g/mL. The pictures show the Mg (green, cement residues) respectively Ca (blue, bone) map dispersion solely.

Possible adhesive residues on bone surfaces were verified via EDS measurements (Figure 4), whereas Mg mainly stems from cement residues and Ca from the bone substrate. A quantitative analysis regarding the Ca-to-Mg-ratio of cement residues and the adherend area is displayed in Table 1. For all cements, initially a calcium: magnesium ratio in the range of ~3.0–7.3 was found on the adherend surfaces, which increased over 7 days to values between 5.6–9.9. Even the latter indicates still a higher amount of Mg^2+^ to be present in the adherend surface (mostly inside the scrub marks which derived from bone substrate preparation, Figure 3A) compared to native bone with a Mg content of ~0.72 wt.% (corresponding to a Ca:Mg ratio of ~55) [28]. Also, whole cement fragments were found in this area for both the newberyite reference and the phytic acid-based adhesives, whereby gradually less cement residues were found on the bone surface after ageing.

## 4. Discussion

Conventional calcium phosphate cements (CPC) are considered to be inappropriate for an application as bone adhesives [15]. Indeed, calcium phosphates (CaP) are described as osteo-inductive materials, which indicates their ability to induce bone formation at heterotopic defect sites [29] and when implanted in cortical bone, a narrow contact between a hydroxyapatite forming CPC and bone was observed [30,31]. However, an instant bone-bonding which would be desirable for their use as bone adhesives is not the case [31]. Barely examples are known in the field of CPC, which directly prove their ability to adhere on bone surfaces. Grover et al. [31] substituted orthophosphoric by pyrophosphoric acid in a brushite forming cement system to improve its bonding on cortical bone and on different biomaterial surfaces [31]. More attempts to enlarge the application field of CaP towards bone adhesives focused on the incorporation of ceramic particles e.g., monocalcium phosphate [19] or β-tricalcium phosphate [18,19,20] into biodegradable methacrylated polylactide-based adhesives [18,19,20]. However, because of their similarity to the mineral phase of bone [15,32], CPC are still promising for applications, where bone-bonding is required [15].

In general, magnesium phosphate cements (MPC) seem to have the higher bonding potential [15] as they were already used in an adhesive manner in vivo for a successfully improved tendon-to-bone-healing [33] and it was shown that MPC are more effective in stabilizing bone fragments to native bone compared to CPC [22]. Gulotta et al. [33] exemplary used a commercially available MPC [20] which was composed of reactive MgO, potassium as well as sodium phosphate and tricalcium phosphate. Though, this formulation has not been approved as bone adhesive so far [15].

In the present study, we used diluted phytic acid as the liquid component of an MPC system to further improve its adhesiveness on bone. The naturally deriving phytic acid is known to strongly bind polyvalent cations as Ca^2+^ [24,25] and Mg^2+^ [25] which are the main constituents of bone and our adhesive. The cement powder mainly consisted of farringtonite, which has recently been demonstrated to react with phytic acid, and 6.0, 6.8 or 7.5 wt.% MgO. The latter was necessary to adjust a proper hardening time as it is a highly reactive source of Mg^2+^ and counteracts the retarding effect that has been observed for phytic acid in combination with mineral bone cements before Christel [23]. Using this approach, it was possible to demold the adhesive-surface constructs already after 10 min and even a fixed porcine cadaver mandible was able to carry its own weight without fracturing after this time (Figure 3B). Measuring the inherent strength of the cements revealed that the setting reaction further proceeds afterwards and reached values around 16−21 MPa for the phytic acid containing samples and 8 MPa for the newberyite reference after 24 h (Figure 1), which is in the range and after longer immersion even superior to the compressive strength of human cancellous bone [34] and similar to MPC obtained by the reaction of farringtonite with acid monocalcium phosphate [35]. While the setting reaction with 2 M phosphoric acid led to a rapid formation of newberyite in the reference cements paste (Figure 2A), no crystalline phases were formed with phytic acid and even prolonged post-curing in water did not lead to a distinct conversion from farringtonite into newberyite as shown by corresponding XRD patterns (Figure 2B).

The adhesive strength determined for our MPCs was initially ~0.75 MPa for the newberyite control and ~1.22 MPa for 25% phytic acid cement. Overall, a mixed adhesive–cohesive failure was observed with remaining cement fragments and particles on the surface and within the scrubs resulting from grinding the bone samples. All samples showed an effect of ageing over a course of 7 d, and the 25% phytic acid adhesive still exceeded the minimal strength of 0.2 MPa necessary for a bone adhesive as proposed by Weber and Chapman [27]. Using a mineral bone cement, Grover et al. [31] observed an initial adhesive strength of 1.3 ± 0.8 MPa on ovine cortical bone which is comparable to what we reached with 25% phytic acid containing samples. We tested the bone-bonding properties on ground bone slices, but did not remove natural impurities as fats and proteins, whereas the bone samples of the as-mentioned publication [31] were defatted prior to bond fabrication. Furthermore, our long-term experiments were performed in a wet and not just humid environment which also can impair the actual bonding strength, but better reflects in vivo conditions. The upper limit of described adhesion to bone is ~14 MPa using a 4-methacryloyloxyethyl trimellitic anhydride/methyl methacrylate adhesive as an interface between human cortical femur and poly(methyl methacrylate) [15,36], but the lack of biodegradability for this system is crucial [15].

## 5. Conclusions

Mineral bone cements are barely tested in vitro for their application as bone-bonding agents and are mostly considered to be non-suitable. In the present study, we showed that a magnesium phosphate cement with unconventional setting mechanism could yet be used as an adhesive on bone, since adhesive strengths of > 0.8 MPa were obtained even after 7 d ageing in a physiological model environment. Although just tested in a proof-of-principle manner with a selected number of experiments, the results indicate that moisture plays a crucial role for the in vitro bonding character. Furthermore, the nature of the adhesive effect has to be investigated in more detail, e.g., is the chelating effect of the phytate ions by its own responsible or is it a combination of the acidic pH of the cements with chelation that improves adhesion. This will help to further improve the described mineral adhesives by design. Although the principal in vitro biocompatibility of phytic acid modified cements has been demonstrated before [37], an application as adhesive must also ensure that the materials will be degraded without impairing bone healing in the fracture gap. However, it is not possible to answer this aspect in pure in vitro experiments as simultaneous material degradation and bone healing is a complicated biological process with a range of different cell types (osteoclast, osteoblasts, macrophages) being involved. After this has been demonstrated, the cements from this study will have the potential to be applied in various clinical scenarios, e.g., midface and orbital fractures or even comminuted cranial fractures. For all cases, only moderate mechanical properties of adhesives are required to arrange small bone fragments into larger structures, which are subsequently fixed by plates and screws to bear mechanical load.

## Figures and Tables

**Table 1:** Ca-to-Mg-ratio on cement and adherend of the different bone adhesive samples. Concentrations of Ca and Mg were calculated based on corresponding EDS element maps. The ratios of one row derive each from the same (border) EDS element map (Figure 4).

		Ca-to-Mg-Ratio (wt.%/wt.%)	
Phytic Acid (%)	Time	Cement Residues	Adherend
*0*	*initial*	0.23 ± 0.02	3.0 ± 0.2
	*24 h*	0.31 ± 0.03	2.7 ± 0.2
	*7 d*	1.6 ± 0.1*	5.6 ± 0.5
*20.0*	*initial*	0.08 ± 0.01	4.9 ± 0.5
	*24 h*	0.20 ± 0.02	4.7 ± 0.2
	*7 d*	0.42 ± 0.05	8.8 ± 0.7
*22.5*	*initial*	0 ± 0	6.2 ± 0.5
	*24 h*	0.17 ± 0.02	8.8 ± 0.9
	*7 d*	0.51 ± 0.04	8.3 ± 0.6
*25.0*	*initial*	0.01 ± 0.01	7.3 ± 0.8
	*24 h*	0.11 ± 0.01	3.9 ± 0.3
	*7 d*	0.26 ± 0.02	9.9 ± 0.8

* barely complete cement residues visible.
